# Uric Acid, Homocysteine, and Inferior Vena Cava Diameter for Early Risk Stratification After Non-ST Elevation Myocardial Infarction

**DOI:** 10.7759/cureus.90952

**Published:** 2025-08-25

**Authors:** Emir Becirovic, Minela Becirovic, Amir Bećirović, Admir Abdic

**Affiliations:** 1 Internal Medicine Clinic, Intensive Care Unit, University Clinical Center Tuzla, Tuzla, BIH; 2 Internal Medicine Clinic, Department of Nephrology, University Clinical Center Tuzla, Tuzla, BIH; 3 Department of Endocrinology and Internal Medicine, University Clinical Center Tuzla, Tuzla, BIH; 4 Department of Surgery, Cantonal Hospital Bihać, Bihać, BIH

**Keywords:** echocardiography, homocysteine, inferior vena cava, myocardial infarction, risk assessment, uric acid

## Abstract

Introduction: Non-ST elevation myocardial infarction (NSTEMI) carries a substantial risk of early major adverse cardiovascular events (MACE) despite advances in therapy. Easily obtainable biochemical and echocardiographic markers may improve early risk stratification, particularly in patients managed without revascularization. This prospective study assessed the prognostic significance of inferior vena cava (IVC) diameter, serum uric acid, homocysteine, and selected hematological indices in predicting 90-day MACE in NSTEMI patients treated with conservative medical therapy. Unlike prior studies that examined these biomarkers individually, our study integrates biochemical (uric acid, homocysteine), echocardiographic (IVC diameter), and hemogram-derived indices into a combined model for early risk stratification in conservatively treated NSTEMI patients.

Methods: A total of 170 consecutive NSTEMI patients admitted to the University Clinical Center Tuzla between February 2022 and January 2023 were included. All patients received guideline-directed medical therapy. Clinical, echocardiographic, and laboratory data were obtained within 24 hours of admission. The primary endpoint was MACE (cardiac death, reinfarction, or urgent coronary revascularization) within 90 days. Logistic regression identified independent predictors; discriminatory ability was assessed using receiver operating characteristic (ROC) analysis, and Kaplan-Meier curves evaluated event-free survival.

Results: MACE occurred in 87 patients (51.2%). Compared to event-free patients, those with MACE had larger IVC diameters (20.25 ± 2.52 mm vs. 18.36 ± 2.16 mm; p < 0.001), higher uric acid (432.8 ± 47.3 μmol/L vs. 358.9 ± 44.6 μmol/L; p < 0.001), and elevated homocysteine levels (18.42 ± 4.13 μmol/L vs. 13.39 ± 2.88 μmol/L; p < 0.001). In multivariate analysis, uric acid (OR per 10 μmol/L = 1.32; 95% CI: 1.05-1.65; p = 0.015) and homocysteine (OR per 1 μmol/L = 1.23; 95% CI: 1.06-1.42; p = 0.005) remained independent predictors. ROC analysis showed excellent discrimination for homocysteine (AUC: 0.844) and uric acid (AUC: 0.830). IVC diameter was associated with lower MACE-free survival (log-rank p = 0.036) but lost significance after adjustment.

Conclusion: Elevated homocysteine and uric acid independently predicted 90-day MACE in NSTEMI patients managed without revascularization. While IVC diameter was not independently predictive, its combination with biochemical markers may enhance risk stratification and guide early post-discharge management. These findings warrant validation in larger multicenter studies.

## Introduction

Acute coronary syndrome (ACS) remains a leading cause of morbidity and mortality worldwide, with non-ST elevation myocardial infarction (NSTEMI) accounting for a substantial proportion of acute presentations [1]. Although associated with lower in-hospital mortality than ST-elevation myocardial infarction (STEMI), NSTEMI carries a clinically significant risk of major adverse cardiovascular events (MACE) in the early post-infarction period, particularly within the first 90 days. This vulnerable phase is marked by a high incidence of reinfarction, cardiac death, and the need for urgent revascularization [2].

Conventional biomarkers, such as high-sensitivity cardiac troponin (hs-cTn) and C-reactive protein (CRP), are central to diagnosis and early risk assessment in ACS; however, their prognostic accuracy for post-discharge adverse events remains limited, particularly in NSTEMI patients treated with conservative medical therapy without revascularization [3]. Additional biochemical and hematological markers that reflect inflammation, oxidative stress, endothelial dysfunction, and prothrombotic states may provide complementary prognostic information. Serum uric acid has been implicated in oxidative stress, endothelial dysfunction, and adverse myocardial remodeling, while elevated homocysteine levels are linked to endothelial injury and accelerated atherosclerosis [4,5]. Both markers have shown independent prognostic value in ACS. Still, no prior study has assessed their combined utility with echocardiographic parameters in NSTEMI patients managed exclusively without percutaneous coronary intervention (PCI) [6].

Among hematological indices, red cell distribution width (RDW-CV), mean platelet volume (MPV), and platelet-large cell ratio (P-LCR) are readily obtainable from routine blood tests and have been variably associated with adverse prognosis in coronary artery disease [7].

Echocardiographic parameters offer additional insights into cardiac structure and hemodynamics. Left ventricular ejection fraction (LVEF) remains the standard measure of systolic function, while regional wall motion abnormalities provide additional insight into ischemic injury and have established prognostic relevance. At the same time, the inferior vena cava (IVC) diameter reflects central venous pressure and intravascular volume status, and the left atrial (LA) diameter serves as a surrogate of chronic diastolic burden [8]. However, few studies have examined these indices together with biochemical markers in NSTEMI cohorts, and none, to our knowledge, have evaluated this specific multimodal combination to enhance early prognostic assessment. The potential of this combined multimodal assessment to significantly enhance early prognostic assessment should instill hope and optimism in the audience. If validated, such an approach could form the basis of a simple, inexpensive, and bedside-applicable tool for identifying high-risk patients during the most vulnerable post-infarction phase [9,10].

This study aimed to evaluate the prognostic value of serum uric acid, homocysteine, RDW-CV, MPV, P-LCR, LVEF, LA diameter, and IVC diameter in predicting 90-day MACE in patients with NSTEMI. We hypothesized that uric acid, homocysteine, and IVC diameter would emerge as the strongest independent predictors in multivariate analysis. By focusing on conservatively treated NSTEMI patients, a population underrepresented in prior studies, this work addresses a clear gap in the literature and proposes a practical multimodal model for early post-infarction risk assessment. The potential impact of these findings on patient care, particularly in the early post-infarction phase, should underscore the importance and relevance of this research to the audience.

## Materials and methods

Study design and setting

This prospective, single-center observational cohort study was conducted from February 2022 to January 2023 at the Intensive Care Unit of the Clinic for Internal Medicine, University Clinical Center Tuzla, which serves as a tertiary referral center for acute coronary syndromes in the region. A total of 170 consecutive patients diagnosed with NSTEMI were included. The primary objective was to evaluate the prognostic value of serum uric acid, homocysteine, RDW-CV, MPV, P-LCR, and IVC diameter in predicting MACE within 90 days. This study represents a secondary analysis of prospectively collected data from patients admitted with NSTEMI to the Clinic for Internal Medicine, University Clinical Center Tuzla. The study protocol was approved by the Ethics Committee of the University Clinical Center Tuzla (Approval No. 02-09/2-97/21; January 12, 2022), and written informed consent was obtained from all participants at the time of initial data collection. Multivariate logistic regression models were adjusted for key demographic and clinical variables, but conventional risk scores such as GRACE (Global Registry of Acute Coronary Events) and TIMI (Thrombolysis in Myocardial Infarction) were not included due to incomplete availability across the cohort.

Patient selection

Our patient selection process was rigorous and thorough. Eligible patients, aged 18 years or older, were required to have NSTEMI confirmed by typical ischemic symptoms, electrocardiographic changes consistent with ischemia, and elevated high-sensitivity troponin I (hs-TnI). We implemented strict exclusion criteria to ensure the reliability and validity of our findings, excluding patients with active infection, autoimmune disease, malignancy, recent trauma or surgery, end-stage renal disease, severe hepatic dysfunction, or myocardial infarction within the preceding 30 days. This approach aimed to reduce confounding effects from non-cardiac conditions that could influence biomarker levels.

Data collection and clinical evaluation

Upon admission, all patients underwent a comprehensive standardized evaluation, including a detailed medical history, physical examination, 12-lead electrocardiography, and venous blood sampling. Complete blood count (CBC) was analyzed using the Sysmex XN-1000 automated hematology analyzer (Sysmex Corporation, Kobe, Japan) to measure platelet indices (MPV, P-LCR) and RDW-CV. Serum hs-TnI, creatinine, glucose, uric acid, CRP, and homocysteine were determined using the Beckman Coulter DxC 700 AU biochemical analyzer (Beckman Coulter Diagnostics, Nyon, Switzerland). All laboratory analyses were performed within 30 minutes of blood collection to ensure parameter stability.

Echocardiographic assessment

Transthoracic echocardiography was performed within 24 hours of admission using a Vivid T8 ultrasound system (General Electric Medical Systems, Jiangsu, China). LVEF was calculated using the biplane Simpson method. LA diameter was measured in the parasternal long-axis view at end-systole. IVC diameter was assessed during spontaneous expiration, 1-2 cm from the right atrial junction, and the mean value from three consecutive cardiac cycles was recorded. All echocardiographic measurements were performed by experienced cardiologists blinded to patient outcomes to reduce observer bias.

Outcome definition and follow-up

The primary outcome was the occurrence of MACE, defined as cardiac death, reinfarction, or urgent coronary revascularization. Patients were followed for 90 days from admission through review of electronic medical records and structured telephone interviews. Event dates were recorded as days from hospital admission.

Statistical analysis

Normality of continuous variables was assessed using the Kolmogorov-Smirnov test. Data are presented as mean ± standard deviation (SD) for normally distributed variables and as median with interquartile range (IQR) for non-normally distributed variables. Categorical variables are expressed as counts and percentages. Between-group comparisons for continuous variables were performed using the Student’s t-test or the Mann-Whitney U test, as appropriate. The chi-squared (χ²) test with Yates’ correction was used for categorical data.

Univariate logistic regression was used to identify potential predictors of 90-day MACE, with results expressed as odds ratios (ORs) and 95% confidence intervals (CIs). For clinical interpretability, ORs for uric acid were calculated per 10 µmol/L increase, for homocysteine per 1 µmol/L increase, and IVC and LA diameters per 1 mm increase. Variables with P < 0.05 in univariate analysis were entered into a multivariate logistic regression model to identify independent associations. Discriminatory performance was evaluated using receiver operating characteristic (ROC) curve analysis, with the area under the curve (AUC) and corresponding 95% CIs calculated for each variable. Differences between AUCs were compared using DeLong’s test. Kaplan-Meier survival analysis was performed to estimate MACE-free survival, stratified by IVC and LA diameter, with differences between survival curves assessed using the log-rank test.

As this was a secondary analysis of pre-existing laboratory and echocardiographic data, all findings should be interpreted as hypothesis-generating. The sample size was determined by the number of eligible patients admitted during the study period, as no a priori power calculation was performed. This exploratory design may have limited the statistical power to detect modest associations, particularly in multivariable models. A two-tailed P-value < 0.05 was considered statistically significant. All statistical analyses were conducted using SPSS version 26.0 (IBM Corp., Armonk, NY) and Python version 3.8 (Python Software Foundation, Wilmington, DE) with the scikit-learn library (version 0.24.2).

## Results

**Figure 1:**
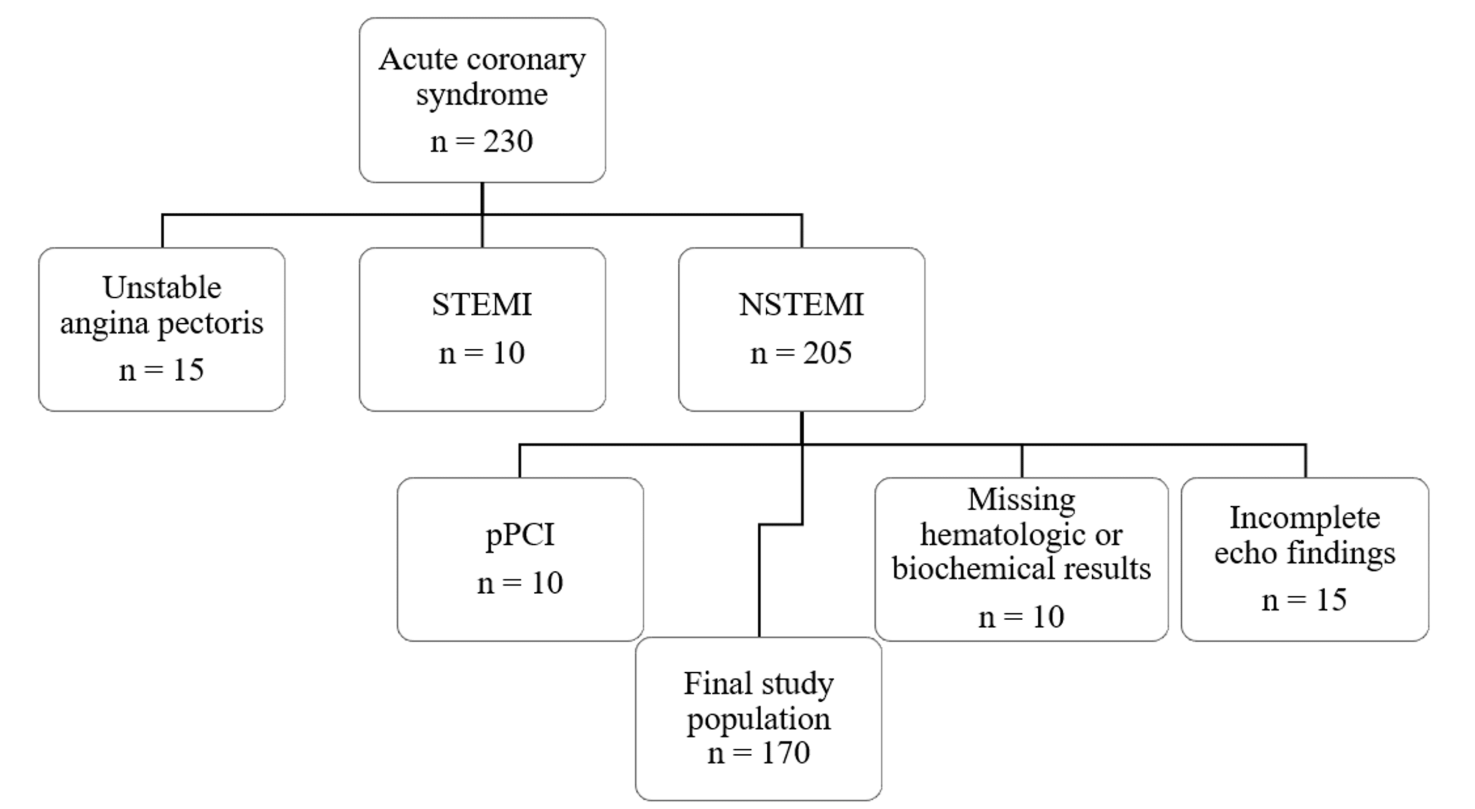
Flowchart of patient selection. Flowchart illustrating patient selection. Of 230 patients admitted with acute coronary syndrome, 15 with unstable angina pectoris and 10 with STEMI were excluded. Among the remaining 205 NSTEMI patients, 10 were excluded due to primary percutaneous coronary intervention, 10 due to missing hematologic or biochemical results, and 15 due to incomplete echocardiographic data. The final study cohort consisted of 170 patients with complete datasets at both time points. STEMI: ST-elevation myocardial infarction; NSTEMI: non-ST elevation myocardial infarction; pPCI: primary percutaneous coronary intervention.

In this prospective, single-center NSTEMI cohort, over half of the patients (51%) experienced a MACE within 90 days, underscoring the high residual risk despite guideline-directed medical therapy. Of the 230 patients admitted with acute coronary syndrome, 15 with unstable angina pectoris (UA) and 10 with STEMI were excluded. Among the 205 patients with NSTEMI, 10 were excluded due to undergoing primary PCI, 10 due to missing hematologic or biochemical results, and 15 due to incomplete echocardiographic data, resulting in a final study cohort of 170 patients with complete datasets at both time points (Figure 1).

The mean age was 68.0 ± 9.8 years in the non-MACE group and 66.1 ± 10.1 years in the MACE group (P = 0.272), and 102 patients (60%) were male. During the 90-day follow-up, 87 patients (51%) experienced a MACE, while 83 patients (49%) remained event-free (Table 1). Baseline characteristics were generally similar between groups, except for a higher prevalence of hypertension (93.1% vs. 81.9%; P = 0.025) and a lower proportion of smokers (42.5% vs. 59.0%; P = 0.032) in the MACE group.

**Table 1:** Baseline characteristics of patients with and without MACE (n = 170). MACE: major adverse cardiovascular events; LA: left atrial; IVC: inferior vena cava; LVEF: left ventricular ejection fraction; RDW-CV: red cell distribution width; MPV: mean platelet volume; P-LCR: platelet-large cell ratio; SD: standard deviation; n: number of patients.

Variable	No MACE (n = 83)	MACE (n = 87)	p-value
Age, years	68.0 ± 9.8	66.1 ± 10.1	0.272
Smoker, n (%)	49 (59.0)	37 (42.5)	0.032
Hypertension, n (%)	68 (81.9)	81 (93.1)	0.025
Diabetes mellitus, n (%)	37 (44.6)	34 (39.1)	0.395
Hyperlipidemia, n (%)	63 (75.9)	64 (73.6)	0.701
Alcohol use, n (%)	43 (51.8)	50 (57.5)	0.485
LA diameter, mm	38.36 ± 3.94	41.16 ± 4.79	<0.001
IVC diameter, mm	18.36 ± 2.16	20.25 ± 2.52	<0.001
LVEF, %	53.34 ± 6.92	49.94 ± 7.34	0.002
RDW-CV, %	13.88 ± 1.30	14.67 ± 1.58	0.003
MPV, fL	9.41 ± 1.34	9.66 ± 1.49	0.218
P-LCR, %	28.96 ± 6.84	30.12 ± 7.43	0.228
Uric acid, μmol/L	358.92 ± 44.62	432.80 ± 47.32	<0.001
Homocysteine, μmol/L	13.39 ± 2.88	18.42 ± 4.13	<0.001

At 24 hours after admission, patients who developed MACE had significantly higher RDW-CV (14.67 ± 1.58% vs. 13.88 ± 1.30%; P = 0.003). Differences in MPV (9.66 ± 1.49 fL vs. 9.41 ± 1.34 fL; P = 0.218) and P-LCR (30.12 ± 7.43% vs. 28.96 ± 6.84%; P = 0.228) were not statistically significant. Biochemical markers were markedly higher in the MACE group, including serum uric acid (432.8 ± 47.3 µmol/L vs. 358.9 ± 44.6 µmol/L; P < 0.001; OR per 10 µmol/L = 1.40; 95% CI: 1.25-1.57) and homocysteine (18.42 ± 4.13 µmol/L vs. 13.39 ± 2.88 µmol/L; P < 0.001; OR per 1 µmol/L = 1.28; 95% CI: 1.15-1.43).

Echocardiography revealed a significantly larger IVC diameter in the MACE group (20.25 ± 2.52 mm vs. 18.36 ± 2.16 mm; P < 0.001; OR per 1 mm = 1.09; 95% CI: 1.02-1.18). LA diameter was also greater in the MACE group (41.16 ± 4.79 mm vs. 38.36 ± 3.94 mm; P < 0.001), while LVEF was lower (49.94 ± 7.34% vs. 53.34 ± 6.92%; P = 0.002). These echocardiographic differences, although statistically significant, were not retained as independent predictors in multivariate analysis (Table 2).

**Table 2:** Univariate and multivariate logistic regression analysis of predictors of MACE. MACE: major adverse cardiovascular events; OR: odds ratio; CI: confidence interval; LA: left atrial; IVC: inferior vena cava; LVEF: left ventricular ejection fraction; RDW-CV: red cell distribution width; MPV: mean platelet volume; P-LCR: platelet-large cell ratio.

Variable	OR (per unit)	95% CI	P-value	Adjusted OR (per unit)	Adjusted 95% CI	Adjusted P-value
LA diameter (mm)	1.02	0.96–1.08	0.538	1.08	0.95–1.22	0.268
IVC diameter (mm)	1.09	1.02–1.18	0.015	1.06	0.87–1.30	0.546
LVEF (%)	0.99	0.96–1.03	0.451	1.04	0.97–1.13	0.270
RDW-CV (%)	0.98	0.91–1.04	0.241	1.00	0.91–1.10	0.941
MPV (fL)	1.00	0.82–1.21	0.991	0.82	0.58–1.15	0.250
P-LCR (%)	1.03	0.97–1.09	0.068	1.01	0.93–1.09	0.768
Uric acid (per 10 µmol/L)	1.40	1.25–1.57	<0.001	1.32	1.05–1.65	0.015
Homocysteine (per 1 µmol/L)	1.28	1.15–1.43	<0.001	1.23	1.06–1.42	0.005

Univariate logistic regression identified uric acid (OR: 1.40 per 10 µmol/L; 95% CI: 1.25-1.57; P < 0.001), homocysteine (OR: 1.28 per 1 µmol/L; 95% CI: 1.15-1.43; P < 0.001), and IVC diameter (OR: 1.09 per 1 mm; 95% CI: 1.02-1.18; P = 0.015) as significant predictors of 90-day MACE, while LA diameter (OR: 1.02; 95% CI: 0.96-1.08; P = 0.538), LVEF (OR: 0.99; 95% CI: 0.96-1.03; P = 0.451), RDW-CV (OR: 0.98; 95% CI: 0.91-1.04; P = 0.241), MPV (OR: 1.00; 95% CI: 0.82-1.21; P = 0.991), and P-LCR (OR: 1.03; 95% CI: 0.97-1.09; P = 0.068) were not statistically significant. These findings are presented with odds ratios and 95% confidence intervals to emphasize the magnitude and precision of the observed associations rather than relying solely on statistical significance.

In the multivariate model, LA diameter (adjusted OR: 1.08; 95% CI: 0.95-1.22; P = 0.268), IVC diameter (adjusted OR: 1.06; 95% CI: 0.87-1.30; P = 0.546), LVEF (adjusted OR: 1.04; 95% CI: 0.97-1.13; P = 0.270), RDW-CV (adjusted OR: 1.00; 95% CI: 0.91-1.10; P = 0.941), MPV (adjusted OR: 0.82; 95% CI: 0.58-1.15; P = 0.250), and P-LCR (adjusted OR: 1.01; 95% CI: 0.93-1.09; P = 0.768) were not independently associated with MACE, while uric acid (adjusted OR: 1.32 per 10 µmol/L; 95% CI: 1.05-1.65; P = 0.015) and homocysteine (adjusted OR: 1.23 per 1 µmol/L; 95% CI: 1.06-1.42; P = 0.005) remained significant. These findings suggest that uric acid and homocysteine, readily available biomarkers, could be integrated into early post-infarction risk stratification models.

**Figure 2:**
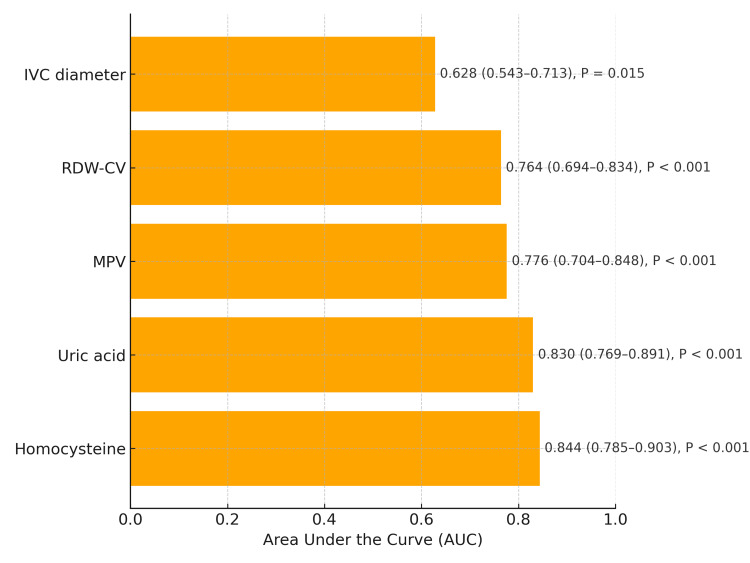
ROC analysis of predictors of MACE. Receiver operating characteristic (ROC) analysis of five clinical variables for predicting major adverse cardiovascular events (MACE) within 90 days after NSTEMI. Homocysteine and uric acid demonstrated the highest discriminative performance (AUC: 0.844 (95% CI: 0.785–0.903) and 0.830 (95% CI: 0.769–0.891), respectively), followed by MPV and RDW-CV. Inferior vena cava diameter showed lower but statistically significant predictive ability. IVC: inferior vena cava; RDW-CV: red cell distribution width; MPV: mean platelet volume; NSTEMI: non-ST elevation myocardial infarction.

ROC analysis showed that homocysteine (AUC: 0.844; 95% CI: 0.785-0.903; P < 0.001) and uric acid (AUC: 0.830; 95% CI: 0.769-0.891; P < 0.001) had the highest discriminative ability, followed by MPV (AUC: 0.776; 95% CI: 0.704-0.848; P < 0.001) and RDW-CV (AUC: 0.764; 95% CI: 0.694-0.834; P < 0.001). IVC diameter demonstrated lower but statistically significant predictive accuracy (AUC: 0.628; 95% CI: 0.543-0.713; P = 0.015) (Figure 2). Optimal thresholds derived from Youden’s index were 15 µmol/L for homocysteine, 400 µmol/L for uric acid, and 19 mm for IVC diameter, providing clinically applicable cut-off values for risk stratification.

Pairwise DeLong tests showed no significant difference between the AUCs of uric acid and homocysteine (P = 0.231). The high AUC values for homocysteine and uric acid indicate robust discriminatory performance, comparable to or exceeding that of several conventional prognostic markers reported in prior NSTEMI studies.

**Figure 3:**
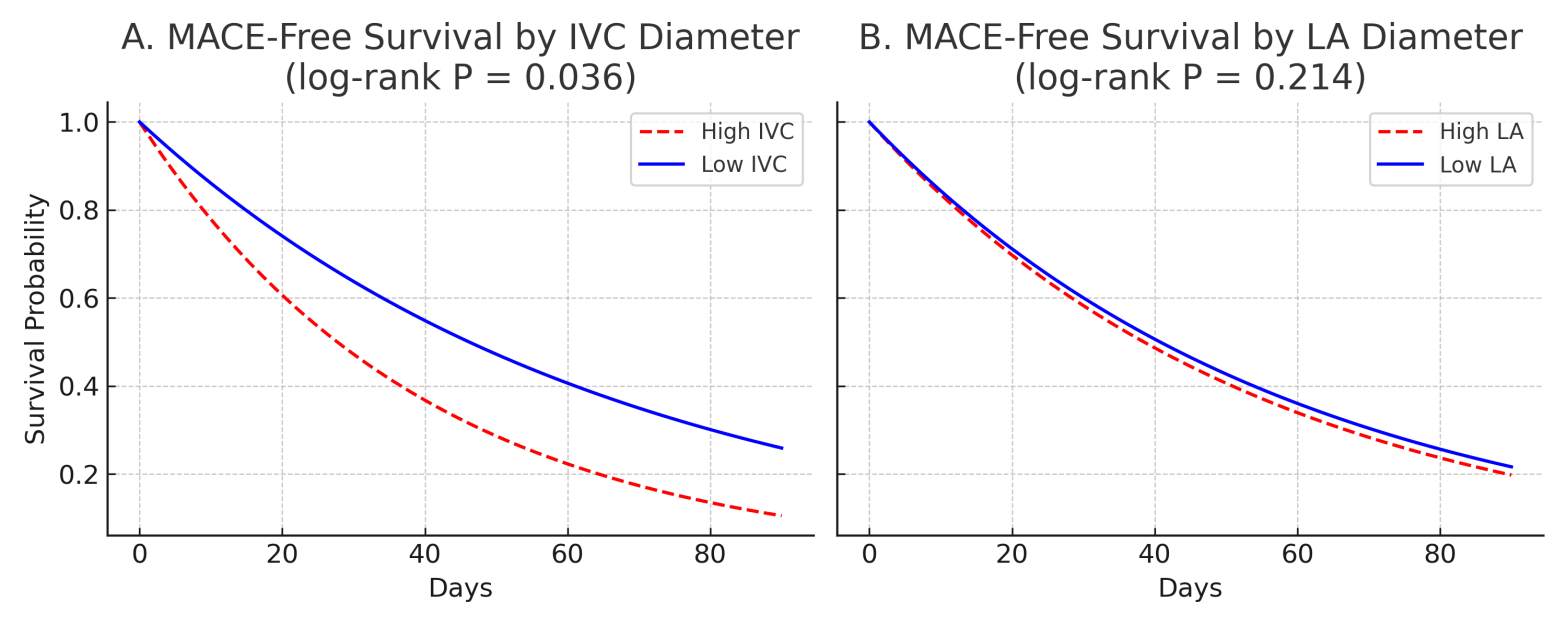
Kaplan–Meier curves for time to MACE according to IVC and LA diameter. Kaplan–Meier survival curves showing 90-day MACE-free survival after NSTEMI. (A) Patients stratified by IVC diameter (high vs. low). The high IVC group demonstrated significantly lower MACE-free survival (log-rank P = 0.036). (B) Patients stratified by LA diameter (high vs. low). No statistically significant difference was observed (log-rank P = 0.214). MACE: major adverse cardiovascular events; LA: left atrial; IVC: inferior vena cava; NSTEMI: non-ST elevation myocardial infarction.

Kaplan-Meier analysis indicated significantly lower MACE-free survival in patients with higher IVC diameters (log-rank P = 0.036), while LA diameter was not significantly associated with outcomes (P = 0.214) (Figure 3). This suggests that although IVC diameter was not independently predictive in multivariate analysis, its association with time-to-event supports its role as a complementary hemodynamic marker in NSTEMI risk assessment.

## Discussion

This study identified serum homocysteine, uric acid, and IVC diameter as significant predictors of 90-day MACE in patients with NSTEMI. In multivariate analysis, only homocysteine and uric acid remained independent predictors, while IVC diameter lost statistical significance after adjustment, suggesting that its prognostic role may be supportive rather than independent. These findings emphasize that easily obtainable, low-cost laboratory measures can offer meaningful prognostic value, especially in healthcare settings where immediate access to advanced imaging or invasive risk stratification is limited. They align with our initial hypothesis that combining biochemical and echocardiographic markers could improve early risk stratification in medically managed NSTEMI [11].

Elevated homocysteine and uric acid levels were strongly associated with adverse outcomes, likely reflecting endothelial dysfunction, oxidative stress, and a heightened prothrombotic state. These mechanisms may accelerate plaque instability, microvascular dysfunction, and thrombogenesis, thereby increasing the likelihood of recurrent ischemic events in the vulnerable early post-infarction phase. [12]. Comparable findings have been observed in a prospective study of 1,306 patients with ACS, where higher discharge homocysteine levels were independently associated with an increased risk of recurrent myocardial infarction during a median follow-up of 9.7 years, supporting our results [13]. Elevated uric acid levels were also identified as an adverse prognostic factor in a cohort of 526 elderly ACS patients, where higher concentrations independently predicted long-term MACE [14]. Our results not only confirm these observations but also extend them to the specific, and underexplored, subgroup of NSTEMI patients treated without PCI, demonstrating their predictive relevance in the early 90-day period. ROC analysis demonstrated excellent discrimination for homocysteine and uric acid (AUC > 0.80), a performance that rivals or exceeds some established clinical scores, supporting their integration into multimodal prognostic models.

Although IVC diameter demonstrated only modest predictive performance (AUC: 0.628) and did not retain significance in multivariate analysis, its value may lie in providing a hemodynamic context to biochemical risk markers. In a cohort of 1,101 patients admitted with acute decompensated heart failure, an IVC diameter >21 mm was independently associated with increased three-year all-cause mortality, particularly in those with renal insufficiency [15]. This consistency with our findings suggests that IVC measurement may be particularly relevant in patients with borderline hemodynamic reserve, serving as an adjunctive tool for identifying those at risk of early decompensation [16]. The link between IVC diameter and outcomes in NSTEMI may reflect impaired venous return, subclinical right heart dysfunction, or elevated filling pressures, which could exacerbate ischemia in patients with limited cardiac reserve.

In our NSTEMI cohort, hematological indices such as RDW-CV, MPV, and P-LCR showed no independent prognostic value, contrasting with findings from a prospective study of 170 NSTEMI patients where neutrophil-to-lymphocyte ratio, platelet-to-lymphocyte ratio, and CRP-to-lymphocyte ratio demonstrated significant predictive potential for MACE [17]. The absence of predictive significance for LA diameter also underscores that, in relatively stable NSTEMI populations, short-term outcomes may be driven more by acute biochemical derangements than by chronic structural changes [18]. Taken together, these observations suggest that a pragmatic model incorporating uric acid, homocysteine, and selected echocardiographic indices could form the basis for future multicenter validation studies. These findings should be considered hypothesis-generating and interpreted with caution until confirmed in larger, prospective cohorts [19].

Limitations

This study has several limitations. It was a single-center observational analysis without randomization, and treatment decisions were made by the attending cardiologists according to current guidelines. Biomarker and echocardiographic measurements were obtained at a single time point, which prevented evaluation of dynamic changes over time. The sample size was limited and not based on an a priori power calculation, which may have reduced the ability to detect modest associations, particularly for RDW-CV, MPV, and P-LCR. Furthermore, GRACE and TIMI risk scores were not incorporated into the multivariate models, as these data were not consistently available for all patients. The follow-up period was restricted to 90 days, and long-term prognostic implications could not be assessed. As this was a secondary analysis, unmeasured confounders cannot be excluded, although the prospective data collection minimized the risk of systematic bias. Therefore, the findings should be interpreted with caution, but their consistency with prior literature supports their potential clinical applicability.

## Conclusions

The independent predictive value of elevated homocysteine and uric acid levels for 90-day MACE in NSTEMI patients managed without revascularization has significant implications for clinical practice. The supportive association of IVC diameter, when combined with biochemical markers, could enhance early risk stratification. Integrating uric acid, homocysteine, and IVC diameter into the initial assessment could provide clinicians with a rapid, inexpensive, and non-invasive tool to identify high-risk patients at the bedside. This could enable earlier intervention during the most vulnerable post-infarction phase. However, larger multicenter studies with extended follow-up are needed to validate and refine this multimodal risk assessment strategy and to determine its utility in diverse healthcare settings.
